# Experimental study of the protective effects of SYVN1 against diabetic retinopathy

**DOI:** 10.1038/srep14036

**Published:** 2015-09-11

**Authors:** Shuo Yang, Heng He, Qi Si Ma, Yong Zhang, Ying Zhu, Xing Wan, Feng Wen Wang, Shuai Shuai Wang, Lei Liu, Bin Li

**Affiliations:** 1 Department of Ophthalmology, Tongji Hospital, Tongji Medical College, Huazhong University of Science and Technology, Wuhan, Hubei Province, China; 2 Department of Optometry and Ophthalmology Center, Tongji Hospital, Tongji Medical College, Huazhong University of Science and Technology, Wuhan, Hubei Province, China; 3 State Key Laboratory Cultivation Base, Shandong Provincial Key Laboratory of Ophthalmology, Shandong Eye Institute, Shandong Academy of medical Sciences, Qingdao, China; 4 Department of Ophthalmology, Taihe Hospital, Hubei University of Medicine, Shiyan, Hubei Province, China

## Abstract

Genetic factors play an important role in the pathogenesis of diabetic retinopathy (DR). While many studies have focused on genes that increase susceptibility to DR, herein, we aimed to explore genes that confer DR resistance. Previously, we identified Hmg CoA reductase degradation protein 1 (SYVN1) as a putative DR protective gene via gene expression analysis. Transgenic mice overexpressing SYVN1 and wild-type (WT) mice with streptozotocin-induced diabetes were used in this experiment. Retinal damage and vascular leakage were investigated 6 months after induction of diabetes by histopathological and retinal cell apoptosis analyses and by retinal perfusion of fluorescein isothiocyanate-conjugated dextran. Compared with diabetic WT mice, diabetic SYVN1 mice had significantly more cells and reduced apoptosis in the retinal ganglion layer. Retinal vascular leakage was significantly lower in diabetic SYVN1 mice than in diabetic WT mice. The expression levels of endoplasmic reticulum (ER) stress-related, pro-inflammatory, and pro-angiogenic genes were also analyzed. Lower expression levels were observed in diabetic SYVN1 mice than in WT controls, suggesting that SYVN1 may play an important role in inhibiting ER stress, chronic inflammation, and vascular overgrowth associated with DR. Thus, these results strongly supported our hypothesis that SYVN1 confers DR resistance.

Diabetic retinopathy (DR) is a major complication of diabetes^1^,^2^; however, clinical data show that not all diabetic patients develop DR. Some diabetic patients are highly susceptible, while others develop only slight abnormalities in the retinal vasculature or none at all. Several factors may lead to these different outcomes, and genetic factors are thought to play a prominent role in susceptibility to DR^3^. Recent studies have focused on the genetic factors that predispose diabetic patients to DR and have identified genes that increase DR susceptibility. However, we adopted the reverse approach and instead focused on identifying genetic factors in diabetic patients who show resistance to developing the vascular complications associated with DR^4^. Some diabetic patients are exposed to risk factors, such as hyperglycemia, for long periods of time without developing DR. We hypothesized that these diabetic patients possess “resistance” genes that protect the retinal vasculature and confer protection against the major retinal complications of diabetes.

In a previous study, we collected blood samples from diabetic patients with or without DR for comparative gene expression analysis^5^. We identified several genetic factors that were correlated with endoplasmic reticulum (ER) stress, such as core/emopamil binding protein (C/EBP), homologous protein (GADD153/CHOP), and glucose-regulated protein 78 (GRP78), and were expressed in diabetic patients who did not develop DR. In a subsequent study, we confirmed that CHOP and other ER stress factors played an important role in the development and progression of DR^6^. To fully elucidate the role of ER stress in the development of DR, we adopted methods previously described by Martin *et al.*^7^. Differences in the gene expression of ER stress factors between hyperglycemic and normal mouse retinas were tested using real-time polymerase chain reaction (PCR) functional gene chips. The results indicated that multiple factors regulating ER-associated degradation (ERAD) were expressed at significantly lower levels in hyperglycemic mouse retinas than in control retinas^8^.

Based on our data, particularly the results of the gene chip experiment, we found that the gene encoding Hmg CoA reductase degradation 1 (SYVN1) may confer resistance to DR. Therefore, we chose to further elucidate the role of this gene in the development and progression of DR. SYVN1 is a core member of the E3 ligase complex in the ERAD pathway. The ERAD pathway clears misfolded and nonfunctional proteins from the ER, thus keeping the ER stable and reducing ER stress. SYVN1 is involved in the recognition, ubiquitination, and disposal of harmful proteins, ensuring that these proteins are transported from the ER and degraded to reduce ER damage. SYVN1 also inhibits apoptosis induced by ER stress^9^,^10^.

In the current study, we generated a transgenic mouse line that overexpresses SYVN1 and used these mice to study the protective role of SYVN1 against DR by inducing diabetes using streptozotocin (STZ) injection. We then directly compared the retinal morphology between SYVN1-overexpressing diabetic mice and wild-type (WT) diabetic mice. Finally, we explored the mechanism of DR resistance in the SYVN1-overexpressing mice by analyzing three potential mechanisms, including inflammatory responses, ER stress pathway activation, and vascular growth factor expression.

## Results

### Generation of SYVN1 mice

**Figure 1:**
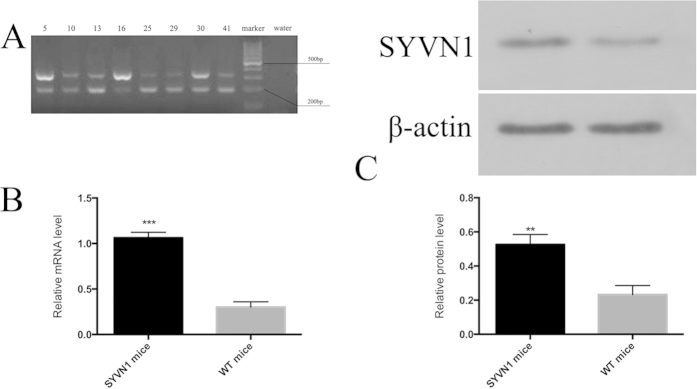
Generation of SYVN1 transgenic mice. (**A**) PCR analysis of *SNVY1* transgene expression. Genomic DNA was extracted from mouse tail samples and amplified by PCR using transgene-specific primers. The PCR product of the *SNVY1* transgene, which encodes SYVN1 protein, was 339 bp. (**B**) Test organs were prepared from SYVN1 transgenic and WT mice. SYVN1 protein was measured by western blotting. β-Actin was used as a protein loading control (n = 3 per group; **p < 0.005). mRNA was measured by RT-PCR (n = 3 per group; ***p < 0.001).

SYVN1 transgenic mice were born healthy with no obvious developmental delays or other defects. They survived to adulthood with high fecundity. SYVN1 transgene expression was confirmed by PCR analysis of the genomic DNA. A transgene-specific, 339-bp DNA fragment was amplified in transgenic mice (Fig. 1A). Compared with WT mice, the transgenic mice had increased SYVN1 expression at both the mRNA (Fig. 1B) and protein levels (Fig. 1C), as observed by RT-PCR (*p* < 0.01) and western blotting (*p* < 0.01).

### Overexpression of SYVN1 reduced vascular leakage in the retina

**Figure 2:**
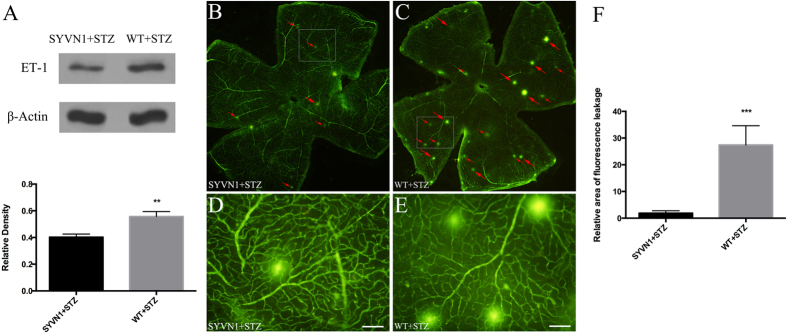
Retinal vascular leakage in diabetic SYVN1 and WT mice. (**A**) Western blot analysis of ET-1 expression in the retina. Results are expressed as the mean ± SD (n = 3 per group; **p < 0.005). (**B**–**E**) Confocal images of retinal flat-mounts ((**B**,**C**) magnified images in (**D**,**E**)). Red arrows depict regions where FITC-conjugated dextran leakage occurred in SYVN1 + STZ (**B**,**D**) and WT + STZ (**C**,**E**) mouse retinas. Scale bar = 100 μm. (**F**) Computer-assisted quantitative analysis of FITC-labeled dextran leakage in diabetic mouse retinas. Results are expressed as the mean ± SD (n = 3 per group; ***p < 0.001). SYVN1 overexpression significantly reduced retinal vascular leakage and ET-1 expression.

Analysis of retinas from diabetic SYVN1 mice showed that vascular leakage was significantly reduced compared with that in retinas from diabetic WT mice, indicating that high SYVN1 expression reduced vascular permeability. Furthermore, the expression of endothelin 1 (ET-1), a genetic marker for vascular damage, was reduced in diabetic SYVN1 mice compared with that in diabetic WT mice. To determine the impact of SYVN1 on retinal microvascular permeability, fluorescein isothiocyanate (FITC)-dextran perfusion was performed in a stretched retinal preparation in diabetic SYVN1 mice, and the retinas were compared with those in diabetic WT mice (Fig. 2). We observed multiple, interspersed regions of leakage of the FITC-conjugated dextran in the diabetic WT mice (Fig. 2C,E), while fewer, less severe leakage points were observed in the diabetic SYVN1 mice (Fig. 2B,D). An analysis of the FITC-dextran leakage areas in the two groups confirmed our observations and showed significantly reduced leakage in the diabetic SYVN1 mice compared with that in the diabetic WT mice (*p* < 0.01). However, the retinal ET-1 level was significantly increased in diabetic WT mice compared with that in diabetic SYVN1 mice (*p* < 0.01).

### Overexpression of SYVN1 protected diabetic mice from retinal damage

**Figure 3:**
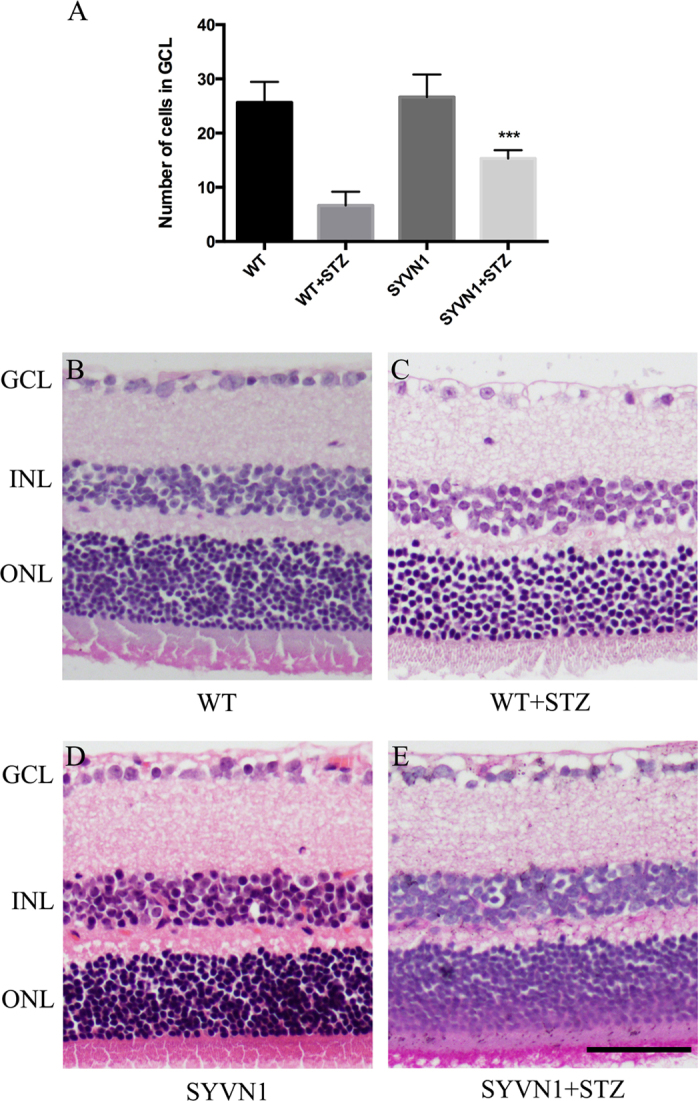
Histopathological analysis of retinas from diabetic SYVN1 and WT mice. (**A**) RGC count in the GCL of retinas harvested from WT, WT + STZ, SYVN1, and SYVN1 + STZ mice. Results are expressed as the mean ± SD (n = 3 per group; ***p < 0.001). (**A**–**D**) Representative images of retinal HE staining. GCL, ganglion cell layer; INL, inner nuclear layer; ONL, outer nuclear layer. Scale bar = 50 μm.

Diabetes can cause pathological changes in the retinal vasculature that can lead to severe structural changes in the retina, including the loss of retinal ganglion cells (RGCs), horizontal cells, amacrine cells, and photoreceptors. Diabetes may also cause expansion of the vascular tissue, disrupting the cell boundary of the inner nuclear layer^11^. Six months after STZ injection, we quantified the number of RGCs in the sectioned retinas of diabetic SYVN1 and WT mice and compared them with retinas in mice administered sodium citrate buffer (non-STZ treated controls). The diabetic SYVN1 mice showed 41.4% fewer RGCs than the non-STZ-treated controls (Fig. 3D), while diabetic WT mice showed 74.4% fewer RGCs (Fig. 3B). Accordingly, there were 56.5% more RGCs in diabetic SYVN1 mice than in diabetic WT mice (*p* < 0.01; Fig. 3E). These results suggested that high SYVN1 expression may prevent apoptosis in RGCs in the outer nuclear layer of retinas from diabetic mice.

**Figure 4:**
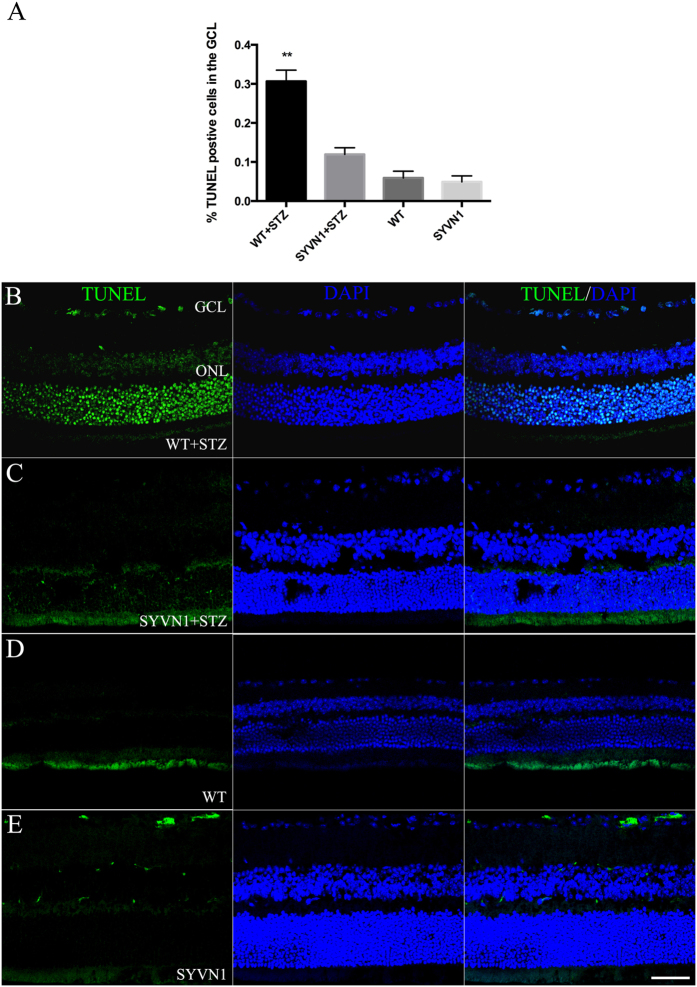
SYVN1 overexpression reduced DNA damage and apoptosis in retinas from diabetic mice and prevented diabetes-induced RBC death. (**A**) Quantification of apoptosis in the retina was determined by counting the number of fluorescently labeled TUNEL-positive cells in the GCL divided by the total number of cells present in the retina. Overexpression of SYVN1 resulted in a lower percentage of TUNEL-positive cells when compared to that in diabetic WT mice. Three sections per retina were imaged and analyzed. Results are expressed as the mean ± SD (n = 3 per group; **p < 0.005). (**B**–**E**) Representative confocal images of retinal TUNEL staining. TUNEL staining in WT + STZ (**B**), SYVN1 + STZ (**C**), WT (**D**), and SYVN1 (**E**) sections.

To explore this hypothesis, we performed TUNEL labeling and quantified the numbers of apoptotic cells in retinas from diabetic WT and SYVN1 mice. Studies have shown that retinal apoptosis occurs as diabetes progresses in mice^12^,^13^. We found that the SYVN1 mice had significantly fewer TUNEL-positive cells than the diabetic WT mice (Fig. 4B,C; *p* < 0.01). Many TUNEL-positive cells were found in the retinal ganglion layer, the inner nuclear layer, and the outer nuclear layer of retinas harvested from diabetic WT mice. In fact, the number of TUNEL-positive cells was significantly decreased by 19% in retinas from diabetic SYVN1 mice compared with that in retinas from diabetic WT mice (Fig. 4A; *p* < 0.01). These results strongly suggested that RGCs were lost due to apoptosis and that SYVN1 protected RGCs from apoptosis in diabetic mice.

### SYVN1 overexpression reduced the expression levels of inflammatory factors, ER stress factors, and vascular endothelial growth factor (VEGF)

**Figure 5:**
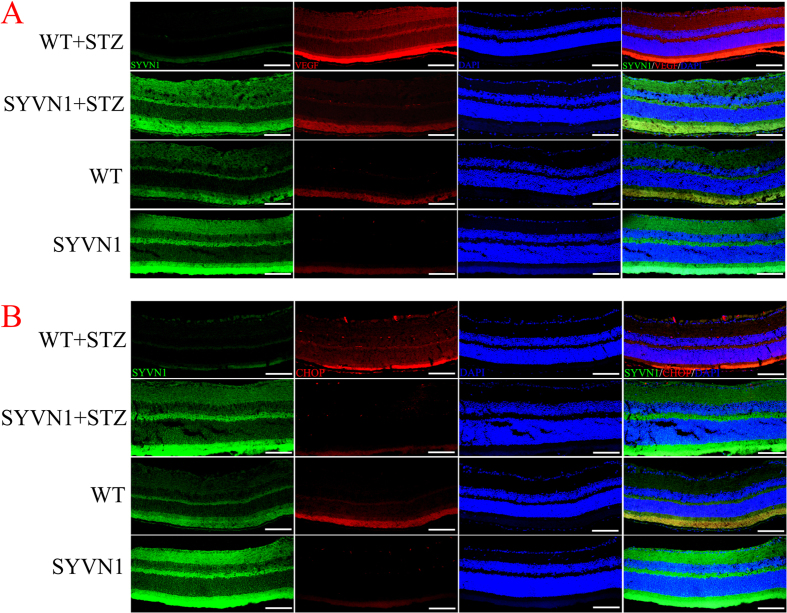
Immunofluorescence staining of retinas from diabetic SYVN1 and WT mice. (**A**,**B**) Immunofluorescence staining for SYVN1 (green)/VEGF (red) and SYVN1 (green)/CHOP (red) in retinal tissue. SYVN1 was labeled with a FITC-conjugated secondary antibody, and VEGF and CHOP were labeled with a Cy3-conjugated secondary antibody. Nuclei were labeled with DAPI (blue). Scale bar = 100 μm.

**Figure 6:**
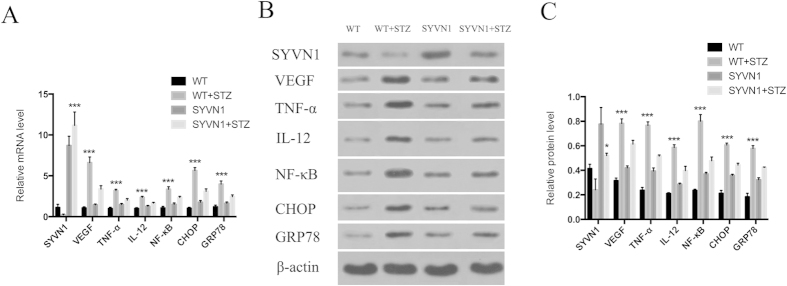
Overexpression of SYVN1 suppressed the expression of VEGF, TNF-α, IL-12, NF-κB, CHOP, and GRP78 in retinas from diabetic mice. (**A**) mRNA levels of *SYVN 1*, *VEGF*, *TNF-α*, *IL-12*, *NF-κB*, *CHOP*, and *GRP78* were determined by real-time PCR. Relative mRNA levels in WT, WT + STZ, SYVN1, and SYVN1 + STZ mice were expressed as the mean ± SD. *SYVN1*: (n = 3; ***p < 0.001 vs. WT + STZ); *VEGF*: (n = 3; ***p < 0.001 vs. WT + STZ); TNF-α: (n = 3; ***p < 0.001 vs. WT + STZ); *IL-12*: (n = 3; ***p < 0.001 vs. WT + STZ); NF-κB: (n = 3; ***p < 0.001 vs. WT + STZ); CHOP: (n = 3; ***p < 0.001 vs. WT + STZ); and GRP78: (n = 3; ***p < 0.001 vs. WT + STZ). (**B**) Representative western blot bands. (C) Protein expression levels were quantified relative to β-actin expression. Relative protein levels of WT, WT + STZ, SYVN1, and SYVN1 + STZ were expressed as the mean ± SD. Full-length blots/gels are presented in Supplementary Figure S1. SYVN1: (n = 3; *p < 0.01 vs. WT + STZ); VEGF: (n = 3; ***p < 0.001 vs. WT + STZ); TNF-α: (n = 3; ***p < 0.001 vs. WT + STZ); IL-12: (n = 3; ***p < 0.001 vs. WT + STZ); NF-κB: (n = 3; ***p < 0.001 vs. WT + STZ); CHOP: (n = 3; ***p < 0.001 vs. WT + STZ); and GRP78: (n = 3; ***p < 0.001 vs. WT + STZ).

Previous studies have shown that the expression levels of VEGF, inflammatory factors, and ER stress factors in the retinas from diabetic mice are significantly increased as the disease progresses^14^,^15^. We reasoned that high levels of SYVN1 expression may negatively regulate VEGF expression and reduce the expression of inflammatory and ER stress factors in the retinas of diabetic mice. We performed immunofluorescence, RT-PCR, and western blot analyses to determine the effects of high SYVN1 expression on the expression levels of these factors. We found that the mRNA and protein expression of VEGF, tumor necrosis factor (TNF)-α, interleukin (IL)-12, nuclear factor kappa B (NF-κB), CHOP, and GRP78 were significantly decreased in the diabetic SYVN1 mice compared with those in diabetic WT mice (*p* < 0.01; Figs 5A,B and 6A–C).

## Discussion

The National Institute of the Human Genome Project found that the majority of human genetic research focuses on identifying genes that predispose to illness^16^. However, a relatively unexplored, but equally important, area of research focuses on the role of genetic factors in maintaining good health. We were inspired by this idea and therefore focused on the gene expression profiles of long-term diabetic patients who did not develop DR. By adopting this approach, we hoped to identify genes that confer resistance to hyperglycemic damage and protect against the retinal vascular complications of DR.

Several previously published reports have demonstrated that ER stress has an important role in many diseases and signaling pathways^17^,^18^,^19^,^20^,^21^. Many reports have shown that ER stress plays a key role in the inflammatory responses and vascular damage that occurs in DR^22^,^23^,^24^,^25^,^26^. VEGF, inflammatory factors, and ER stress factors have been shown to interact during the progression of DR^24^. ER stress pathways are closely linked to inflammatory pathways, and the expression levels of VEGF and inflammatory factors are upregulated under conditions of ER stress^27^. ER stress has also been shown to activate the apoptotic pathway, leading to apoptosis of RGCs and pericytes^17^,^28^,^29^,^30^,^31^,^32^.

In several previous studies, we investigated a series of factors that may inhibit ER stress in DR^5^,^6^,^33^,^34^. ERAD is important in ER homeostasis. ER stress is inhibited by a reduction in the accumulation of harmful proteins in the ER^10^,^35^,^36^. Under hyperglycemic conditions caused by diabetes, the internal ER environment is altered, causing ER stress, which leads to anomalies in transcriptional regulation, ion channel function, metabolism, and signal transduction^19^,^37^,^38^,^39^. SYVN1 plays a key role in ERAD^40^,^41^, functioning to identify unfolded or misfolded proteins in the ER lumen and shuttle them to the cytoplasm for degradation, thereby reducing ER stress and apoptosis^42^. In studies of degenerative diseases of the nervous system, SYVN 1 has been shown to reduce ER stress, thus preventing apoptosis of neural cells^43^,^44^,^45^.

We previously reported that SYVN1 expression was downregulated in diabetic mouse retinal tissue. Its expression was also reduced *in vitro* in retinal vascular endothelial cells that were cultured in high-glucose media^8^. This caused the attenuation of ERAD function and the accumulation of unfolded proteins in the ER, which produced ER stress. In addition to apoptosis, ER stress can induce inflammatory responses through several mechanisms^27^, and these chronic inflammatory responses can promote the development and progression of DR^23^,^44^,^46^. The current study focused on the effects of SYVN1 in the retinas of diabetic mice and on the role of related factors in three pathways, i.e., inflammatory factors (TNF-α, IL-12, and NF-κB), ER stress factors (CHOP and GRP78), and vascular growth factors (VEGF).

VEGF is a prominent vascular growth factor and is implicated in the development of DR. An increase in VEGF expression can significantly increase vascular permeability^47^ and upregulate the expression of certain adhesion molecules, causing damage to vascular endothelial cells^46^. NF-κB is a transcription factor that is induced by TNF-α, which itself plays an important role in regulating inflammation, immune responses, cell proliferation, and apoptosis. TNF-α also participates in the pathological progression of DR^47^,^48^,^49^,^50^,^51^. NF-κB activation occurs in both human diabetic patients and animal models of diabetes^52^,^53^. Animal studies have shown that inhibition of NF-κB reduces retinal vascular damage and cell death caused by diabetes^54^. CHOP is a transcription factor that is involved in ER stress. It binds to the promoter of the *TRB3* gene to suppress Akt activation, which can cause apoptosis^55^,^56^. The protein kinase RNA-like endoplasmic reticulum kinase (PERK)-CHOP pathway is involved in ER stress and is linked to ganglion cell apoptosis^57^,^58^. GRP78 is a marker for ER stress response^18^,^23^,^59^,^60^ and may be involved in retinal neovascularization in DR^61^. Our results demonstrated that the expression levels of these important factors were significantly lower in the retinas of diabetic SYVN1 mice than in those of diabetic WT mice. Furthermore, we found that as DR developed, SYVN1 overexpression significantly reduced the levels of inflammatory cytokines, ER stress factors, and VEGF. When interpreted in conjunction with our analysis of retinal morphology, retinal vascular permeability, and RGC apoptosis, we showed that SYVN1 reduced retinal damage and may protect diabetic patients against the development and progression of DR.

Increased vascular permeability is a striking feature of early-stage DR^1^,^2^. Our study found that vascular permeability was reduced in diabetic SYVN1 transgenic mice compared with that in diabetic WT mice, suggesting that SYVN1 may confer resistance against increases in retinal vascular permeability caused by hyperglycemia. We also found that ET-1 protein expression, an indicator of retinal vascular damage, was reduced in diabetic SYVN1 mice compared with that in diabetic WT mice. RGC apoptosis is also a pathological consequence of DR^1^,^2^. We observed that the morphology and organization of the retinal layers was more intact and less disorganized in diabetic SYVN1 mice than in diabetic WT controls. Furthermore, vascular hyperplasia was reduced, while cells in the nerve cell layer were significantly increased in diabetic SYVN1 mice compared with that in diabetic WT mice. The number of apoptotic cells was significantly lower in the retinas of mice that expressed high levels of SYVN1 compared with that in controls. Taken together, our results strongly suggested that SYVN1 functioned as a DR-resistance gene. In our study, the transgenic mice exhibited global overexpression of SYVN1. Therefore, based on this model, secondary effects from the global overexpression may complicate the results, potentially causing the changes observed in the expression levels of VEGF, inflammatory factors, and ER stress factors between the transgenic and WT mice. There are several potential explanations for these changes. Primarily, alterations in signaling pathways and interactions caused by high expression of *SYVN1*, which encodes SYVN1, may cause changes in these factors. Further studies are required to determine the interactions between various signaling pathways. Additionally, we found that the expression levels of NF-κB, CHOP, and GRP48 differed between transgenic and WT mice. These observations may be explained by the complexity of organisms, in which various signaling pathways may influence each other, thereby regulating the expression levels of these genes. Alternatively, high expression of the target gene may affect these various signaling pathways. However, despite this limitation, our results provide important insights into new strategies for the prevention and treatment of DR in patients with diabetes.

## Research Design and Methods

### Animals

The genetic background of the mice used in this experiment, including WT and transgenic mice, was C57bl/6N. All animal studies were carried out in accordance with the NIH Guidelines for the Care and Use of Laboratory Animals. All mice were bred at the Experimental Animal Center of Tongji Medical College, Huazhong University of Science and Technology, China in specific pathogen-free (SPF) conditions. All mouse experiments were reviewed and approved by the Ethics Committee for Animal Care and Use of Tongji Medical College, China.

### Generation, breeding, and analysis of SYVN1 mice

The novel mouse line, pRP -SYVN1, was generated to express high levels of the *SYVN1* gene. To generate genetically modified mice, the target gene construct was synthesized through extraction, enzyme digestion, and purification of the plasmid. This construct was then microinjected into fertilized eggs (Cyagen Biosciences, Guangzhong, China). Total DNA was extracted from tail tissue samples of the genetically modified F_0_ offspring. Transgene-specific primers were used for PCR amplification of the SYVN1 construct as follows: forward, 5′-CTCAAGCCTCAGACAGTGGTT-3′ and reverse, 5′-AACAGCGTACCAGGACCGTTC-3′.

PCR amplification was performed in triplicate under the following conditions: 3 min at 94 °C; 35 cycles of 94 °C (30 s), 52 °C (49 s), and 72 °C (30 s); and a final 10-min incubation at 72 °C. Western blotting and RT-PCR were used to confirm the expression of RNA and protein. *SYVN1* transgene-positive mice were chosen for subsequent procedures.

### Diabetic mouse model

WT (n = 30) and SYVN1 (n = 30) mice (8–9 weeks of age) received an intraperitoneal injection of streptozotocin (STZ; 50 mg/kg) for 5 consecutive days to induce diabetes. Weight- and age-matched nondiabetic control mice (WT, n = 30; SYVN1, n = 20) received an intraperitoneal injection of sodium citrate buffer (50 mg/kg) for 5 days. Mice were provided food and water ad libitum. Seven days after injection, the blood glucose level was measured using a blood glucose meter (Johnson & Johnson, USA) in blood collected from the vena caudalis. The mean blood glucose level of the diabetic mice was 15.00 mmol/L, and the mean blood glucose level of control mice remained steady at 5.37 mmol/L. Weight and blood glucose levels were measured monthly for 6 months, and the mice were used in subsequent experiments.

### Retinal analysis

A flat-mount retinal preparation after FITC-dextran perfusion was used to visualize vascular leakage in mouse retinas (one eye per mouse and three mice per group). Mice were euthanized with sodium pentobarbital (45 mg/kg) by intraperitoneal injection. The mice were placed in the supine position, and 0.3 mL of a high-molecular-weight FITC-conjugated dextran solution (2 × 10^6^; 25 mg/mL; Sigma, USA) was perfused via the superior vena cava. Five minutes after intravital circulation, the eyes were enucleated, immediately placed in paraformaldehyde solution (40 g/L), and fixed for 40 min. The retinas were carefully dissected using forceps and flat-mounted onto glass slides. Retinal vessels were visualized under an Olympus BX51 fluorescence microscope (Olympus America, Center Valley, PA, USA), and retinal vascular leakage was analyzed using ImageJ software (National Institutes of Health, Bethesda, MD, USA).

The retinal vasculature was also histologically analyzed. Mice were euthanized with an overdose (90 mg/kg) of sodium pentobarbital. The eyes were enucleated and immediately placed in paraformaldehyde solution (40 g/L) for 24 h. The eyes were then dehydrated using a graded ethanol series and embedded in paraffin. The whole eyes were serially sectioned (5-μm thick) along the vertical meridian and stained with hematoxylin and eosin (HE; Hubei BIOS Bio-tech Co.; Ltd.). One eye per mouse and three mice per group were analyzed. Three sections from each eye at the same position were stained for analysis using ImageJ software (National Institutes of Health, Bethesda, MD, USA).

Retinal cell apoptosis and DNA damage were measured by the transferase-mediated dUTP nick-end labeling (TUNEL) assay. Mice were euthanized with an overdose of sodium pentobarbital. The eyes were enucleated and immediately placed in paraformaldehyde solution (40 g/L) overnight. The eyes were then dehydrated for 8 h using 30% sucrose solution, and the anterior segment and vitreous body were removed. The eyes were embedded in Tissue-Tek OCT medium (Sakura-Finetek, Japan, Tokyo, Japan) and serially cryo-sectioned (10 μm). One eye per mouse and three mice per group were analyzed. Three sections from each eye at the same position were labeled using a TUNEL kit according to the manufacturer’s instructions (Roche, Mannheim, Germany). The labeled retinas were visualized by confocal laser-scanning microscopy (Nikon Eclipse Ti-SR; Japan) and analyzed using ImageJ software (National Institutes of Health).

Retinal tissue and frozen sections were obtained as described above. Immunofluorescent labeling was carried out as previously described, with minor modifications^8^. Immunofluorescent double labeling was performed for VEGF and CHOP after staining sections for SYVN1. The following primary antibodies were used: anti- SYVN1 (1:100; bs-0679R; Biosynthesis Biotechnology, Beijing, China), anti-VEGF (1:200; ab1316; Abcam, USA), and anti-CHOP (1:150; BS1527; Bioworld). The sections were incubated in the primary antibody solution at 4 °C overnight, washed, and labeled with Cy3- or FITC-conjugated secondary antibodies (1:100; BA1105, BA1031; Boster, Wuhan, China). The nuclei were stained with DAPI. The labeled sections were visualized by confocal laser-scanning microscopy (Nikon Eclipse Ti-SR; Nikon, Tokyo, Japan).

### RT-PCR

Changes in inflammatory and vascular-related gene expression in the retina were determined by RT-PCR. Mice were euthanized by intraperitoneal injection of pentobarbital sodium (45 mg/kg; n = 3 per group). The eyes were rapidly enucleated, and the retinas were dissected. Total RNA was extracted from retinas using an RNA Extraction Kit (E.Z.N.A. Total RNA Kit; OMEGA, USA), and cDNA was generated using the RevertAid First Strand cDNA Synthesis Kit (Fermentas, Lithuania) according to the manufacturer’s instructions. Relative mRNA levels were measured according the 2^−ΔΔCt^ method following MIQE guidelines^62^. Gene-specific primer sequences and annealing temperatures are shown in Table 1.

### Western blot analysis

Changes in inflammatory and vascular-related protein expression in the retina were determined by western blot analysis. Retinal tissue was isolated as described above. Western blot analysis was performed as previously described^8^. The following primary antibodies were used: anti- SYVN1 (1:500; bs-0679R; Biosynthesis Biotechnology), anti-VEGF (1:300; ab1316; Abcam), anti-CHOP (1:600; BS1527; Bioworld), anti-TNF-α (1:600; ab1316; Abcam), anti-GRP78 (1:600; BS1154; Bioworld), anti-NF-κB (1:1000; sc-8008; Santa Cruz Biotechnology), anti-IL-12 (1:300; sc-74147; Santa Cruz Biotechnology), and anti-β-actin (1:600; BM0627; Boster). The following secondary antibodies were used: HRP-labeled sheep anti-mouse (1:50000; BA1051; Boster) and HRP-labeled goat anti-rabbit (1:50000; BA1054, BA1031; Boster). The retinal tissue was incubated in the primary antibodies overnight and the secondary antibodies for 2 h.

### Statistical analysis

Normally distributed data were statistically analyzed with the independent two-sample *t*-test or one-way ANOVA using SPSS Statistics15.0 software (SPSS Inc., Chicago, IL, USA). Data were presented as the mean ± standard deviation (SD). Differences with *p*-values of less than 0.05 were considered statistically significant.

## Additional Information

**How to cite this article**: Yang, S. *et al.* Experimental study of the protective effects of SYVN1 against diabetic retinopathy. *Sci. Rep.* **5**, 14036; doi: 10.1038/srep14036 (2015).

## Supplementary Material

Supplementary Information

## Figures and Tables

**Table 1:** Real-Time-PCR primers used to assess gene expression in mouse tissue.

Target gene	Primers (5′→3′)	Annealing temperature
SYVN1	F: CTTCGTCAGCCACGCTTATC	60 °C
	R: CCACGGAGTGCAGCACATAC	
VEGF	F: GCTACTGCCGTCCGATTGAG	60 °C
	R: GCTGGCTTTGGTGAGGTTTG	
TNF-α	F: CGTCAGCCGATTTGCTATCT	60 °C
	R: CGGACTCCGCAAAGTCTAAG	
IL-12	F: CGAAACCTGCTGAAGACCAC	60 °C
	R: AGCTCCCTCTTGTTGTGGAA	
NF-κB	F: TGCTGGAAGTCACATCTGGT	60 °C
	R: TGCTGAGGATTCTGTCGTGT	
CHOP	F: TCACTACTCTTGACCCTGCG	60 °C
	R: ACTGACCACTCTGTTTCCGT	
GRP78	F: TCTCAGATCTTCTCCACGGC	60 °C
	R: CTTCAGCTGTCACTCGGAGA	
β-actin	F:CACGATGGAGGGGCCGGACTCATC	60 °C
	R: TAAAGACCTCTATGCCAACACAGT	
