# Supplementary material for: Experimental study of the protective effects of SYVN1 against diabetic retinopathy
Source: Sci Rep. 2015 Sep 11;5:14036. doi: 10.1038/srep14036 (PMC4642554; doi:10.1038/srep14036)

**Experimental study of the protective effects of SYVN1 against diabetic retinopathy**

Short Title: SYVN1 protects against diabetic retinopathy

Shuo Yang^1,2,3^, Heng He^1^, Qi Si Ma^1^, Yong Zhang^4^, Ying Zhu^1^, Xing Wan^1^, Feng Wen Wang^1^, Shuai Shuai Wang,^1^ Lei Liu^2^, Bin Li^1^*

^1^Department of Ophthalmology, Tongji Hospital, Tongji Medical College, Huazhong University of Science and Technology, Wuhan, Hubei Province, China

^2^Department of Optometry and Ophthalmology Center, Tongji Hospital, Tongji Medical College, Huazhong University of Science and Technology, Wuhan, Hubei Province, China

^3^State Key Laboratory Cultivation Base, Shandong Provincial Key Laboratory of Ophthalmology, Shandong Eye Institute, Shandong Academy of medical Sciences, Qingdao, China

^4^Department of Ophthalmology, Taihe Hospital, Hubei University of Medicine, Shiyan, Hubei Province, China

***Corresponding Author:** Bin Li

Department of Ophthalmology, Tongji Hospital, Tongji Medical College, Huazhong University of Science and Technology

1095 Jie-fang Road, Wuhan, Hubei Province, People’s Republic of China

Phone: +86-13638673626

Fax: +86-2783663223

Email: [libin-12@163.com](mailto:libin-12@163.com)

**Supplementary Materials**

**Figure S1** The expression of SYVN1, VEGF, TNF-α, IL-12, NF-κB, CHOP, and GRP78 in retinas from every group.

**Raw cropped gels/blots**

Our experiments were performed under the same experimental conditions (including experimental procedure, Operator, instrument etc.) In our text we selected the representative western blots below. So we list the complete images here. （In the picture, HRD1 and SYVN1 are different names for the same protein.）


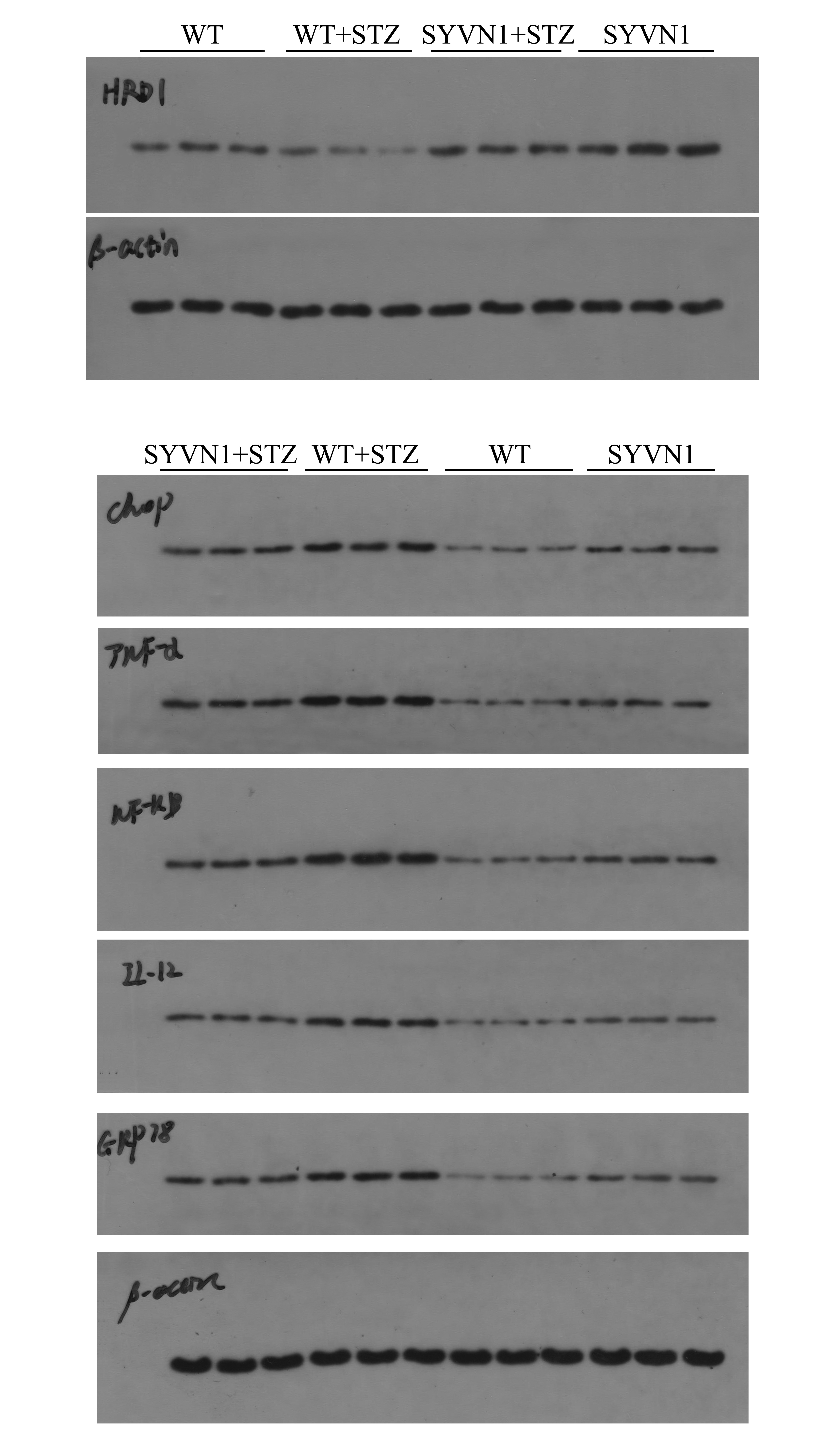

Supplement: Supplementary Information [file srep14036-s1.doc]
